# Neutrophil-mediated delivery of the combination of colistin and azithromycin for the treatment of bacterial infection

**DOI:** 10.1016/j.isci.2022.105035

**Published:** 2022-08-30

**Authors:** Jiacong Gao, Xueyan Hu, Congjuan Xu, Mingming Guo, Shouyi Li, Fan Yang, Xiaolei Pan, Fangyu Zhou, Yongxin Jin, Fang Bai, Zhihui Cheng, Zhenzhou Wu, Shuiping Chen, Xinglu Huang, Weihui Wu

**Affiliations:** 1 State Key Laboratory of Medicinal Chemical Biology, Key Laboratory of Molecular Microbiology and Technology of the Ministry of Education, Department of Microbiology, College of Life Sciences, Nankai University, Tianjin 300071, China; 2 Key Laboratory of Bioactive Materials for the Ministry of Education, College of Life Sciences, State Key Laboratory of Medicinal Chemical Biology, Nankai University, Tianjin 300071, China; 3 Joint Laboratory of Nanozymes, College of Life Sciences, Nankai University, Tianjin 300071, China; 4 College of Life Sciences, Nankai University, Tianjin 300071, China; 5 Department of Laboratory Medicine, 5th Medical Center of Chinese PLA General Hospital, Beijing, China

**Keywords:** Immunology, microbiology, bacteriology

## Abstract

Novel treatment strategies are in urgent need to deal with the rapid development of antibiotic-resistant superbugs. Combination therapies and targeted drug delivery have been exploited to promote treatment efficacies. In this study, we loaded neutrophils with azithromycin and colistin to combine the advantages of antibiotic combinations, targeted delivery, and immunomodulatory effect of azithromycin to treat infections caused by Gram-negative pathogens. Delivery of colistin into neutrophils was mediated by fusogenic liposome, while azithromycin was directly taken up by neutrophils. Neutrophils loaded with the drugs maintained the abilitity to generate reactive oxygen species and migrate. *In vitro* assays demonstrated enhanced bactericidal activity against multidrug-resistant pathogens and reduced inflammatory cytokine production by the drug-loaded neutrophils. A single intravenous administration of the drug-loaded neutrophils effectively protected mice from *Pseudomonas aeruginosa* infection in an acute pneumonia model. This study provides a potential effective therapeutic approach for the treatment of bacterial infections.

## Highlights

• Neutrophils are loaded with colistin and azithromycin *in vitro*
• The loaded drugs enhance the bactericidal effect and reduce the inflammatory response
• Drug-loaded neutrophils conferred effective protection against bacterial infection

## Graphical abstract

**Figure:**
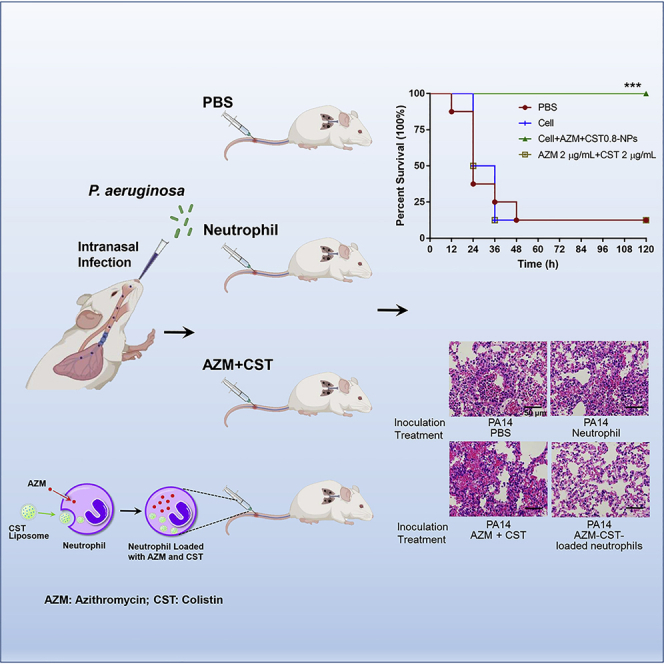

## Introduction

The growing antibiotic resistance poses a severe threat to human health. In 2017, the World Health Organization (WHO) announced a list of antibiotic-resistant pathogens for which new antibiotics are urgently needed. Particularly, carbapenem-resistant *Acinetobacter baumannii*, *Pseudomonas aeruginosa,* and Enterobacteriaceae (including *Klebsiella pneumoniae*, *Escherichia coli*, *etc*.) were listed as the most critical group.

*P. aeruginosa* is an opportunistic pathogen that mainly causes nosocomial infections in patients with compromised immunity, burn wound, diabetic foot, and cystic fibrosis (CF) (Juan et al., 2017). The bacterium is intrinsically resistant to a wide range of antibiotics owing to low membrane permeability, multiple drug efflux systems, and chromosomally encoded antibiotic modifying enzymes, such as the β-lactamase AmpC, *etc* (Lister et al., 2009). Mutations that alter drug targets, reduce membrane permeability, or increase the expression of efflux pumps further increase the resistance. In addition, horizontal acquisition of antibiotic-resistant genes, such as carbapenemases plays important role in antibiotic resistance in clinical settings.

In chronic infections, bacteria usually form biofilms that greatly enhance bacterial tolerance to antibiotics and host immune clearance (Taylor et al., 2014). Biofilms are bacterial cells embedded in an extracellular matrix composed of exopolysaccharides, extracellular DNA, and proteins, which serve as physical and chemical shields against antimicrobial substances and immune cells (Thi et al., 2020; Soares et al., 2020). Previous studies revealed that *P. aeruginosa* biofilm was surrounded by polymorphonuclear (PMN) cells in CF lungs (Alhede et al., 2014; Bjarnsholt et al., 2009). However, failure to eradicate the biofilm results in the continuous recruitment of PMNs and chronic inflammation, leading to the deterioration of lung function (Alhede et al., 2014; Bjarnsholt et al., 2009). Therefore, the combination of antimicrobial and anti-inflammatory strategies may improve therapeutic efficacies and prognoses in patients infected with *P. aeruginosa* or other bacteria.

Azithromycin is a macrolide antibiotic with anti-inflammatory properties (Amsden, 2005). In addition, azithromycin has been shown to inhibit the biofilm formation and production of multiple virulence factors by suppressing the quorum sensing (QS) systems of *P.* *aeruginosa* (Parnham et al., 2014; Seleem et al., 2020). However, azithromycin displays high minimum inhibitory concentrations (MICs) for *P. aeruginosa*, ranging from 8 to 512 μg/mL or higher (Leroy et al., 2021). Combinations of azithromycin with other antibiotics, such as antimicrobial peptides, ciprofloxacin, gentamicin, and colistin have been shown to confer synergistic therapeutic effects against bacterial infections (Liu et al., 2020; Lin et al., 2015; Saini et al., 2015). Colistin (polymyxin E) and polymyxin B represent the last resort against Gram-negative pathogenic bacteria resistant to essentially all other antibiotics. However, the side effects of polymyxins, especially nephrotoxicity and neurotoxicity limit clinical usage (Fiaccadori et al., 2016). Therefore, local delivery of polymyxins would be beneficial for the treatment.

Neutrophils are the most abundant phagocytic cells that are among the earliest leukocytes to reach the infection site, making them an ideal drug delivery vector to treat bacterial infections (Chu et al., 2018). In addition, neutrophils released antimicrobial substances and generated reactive oxygen species (ROS) might synergize with antibiotics in bacteria killing.

**Figure 1:**
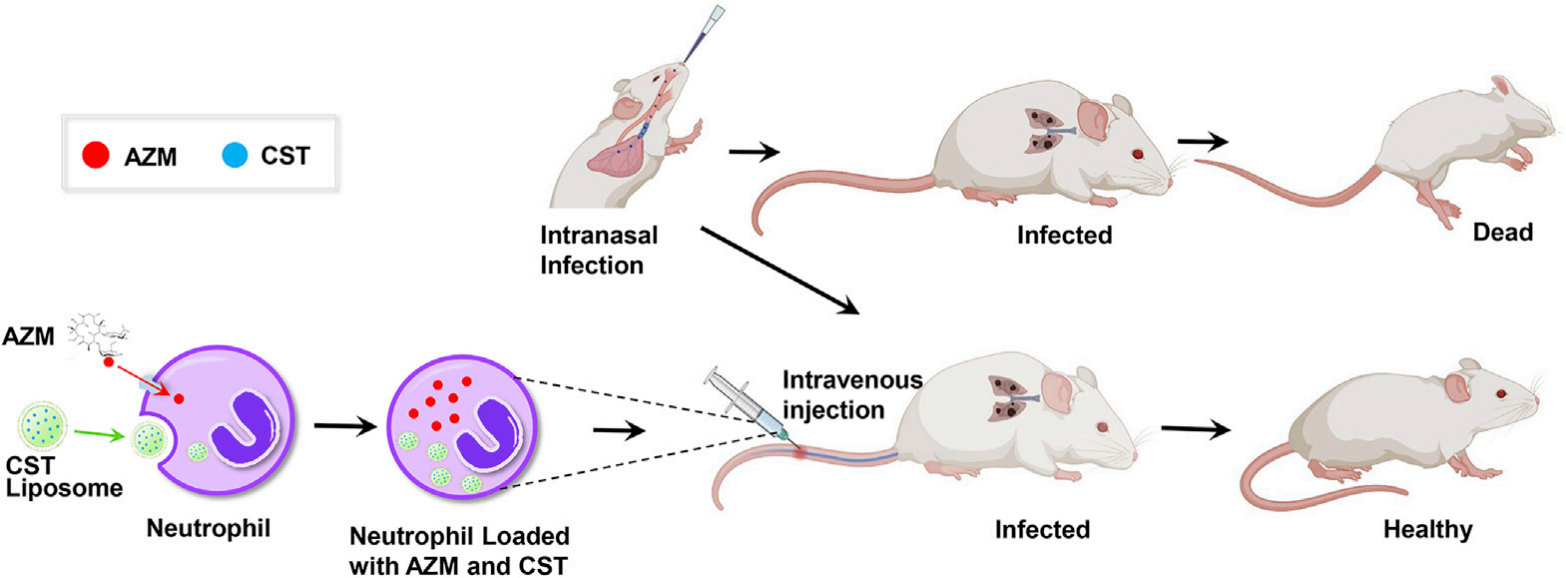
Schematic illustration of azithromycin and colistin loaded neutrophils for the effective treatment of bacterial infection in a mouse acute pneumonia model Azithromycin is taken up directly by neutrophils. Intracellular delivery of colistin is mediated by colistin loaded liposomes. Mice are infected by *P. aeruginosa* intranasally and neutrophils are administered through tail vein injection. AZM, azithromycin; CST, colistin.

In this study, we aimed to develop “super-neutrophils” as an effective therapeutic system against infections caused by Gram-negative pathogens, including “superbugs.” We loaded neutrophils with azithromycin and colistin to combine the following advantages: (1) quick recruitment of neutrophils to the infection site (Zhang et al., 2009); (2) efficient bactericidal activity of colistin and currently low-resistant rates of clinical Gram-negative pathogens (Antonucci et al., 2014; Mohamed et al., 2016); (3) synergistic bactericidal effect between colistin and azithromycin (Bae et al., 2016); (4) immunomodulatory and anti-bacterial virulence activities of azithromycin (Pohl et al., 2020); (5) stable retaining of the drugs in neutrophils in the absence of pathogens (Vazifeh et al., 1997); (6) simultaneous delivery of the drugs to overcome pharmacokinetic difference (Mayer et al., 2019). Azithromycin is naturally accumulated in neutrophils (Wilms et al., 2006; Hand and Hand, 2001). Colistin was nano-packaged into fusogenic liposomes to promote neutrophil uptake. Our *in vitro* and *in vivo* results demonstrate the enhanced bactericidal effect and reduced inflammatory response of the drug-loaded neutrophils, indicating that this system may serve as an efficient strategy to treat bacterial infections (Figure 1).

## Results

### Accumulation of azithromycin in neutrophils

**Figure 2:**
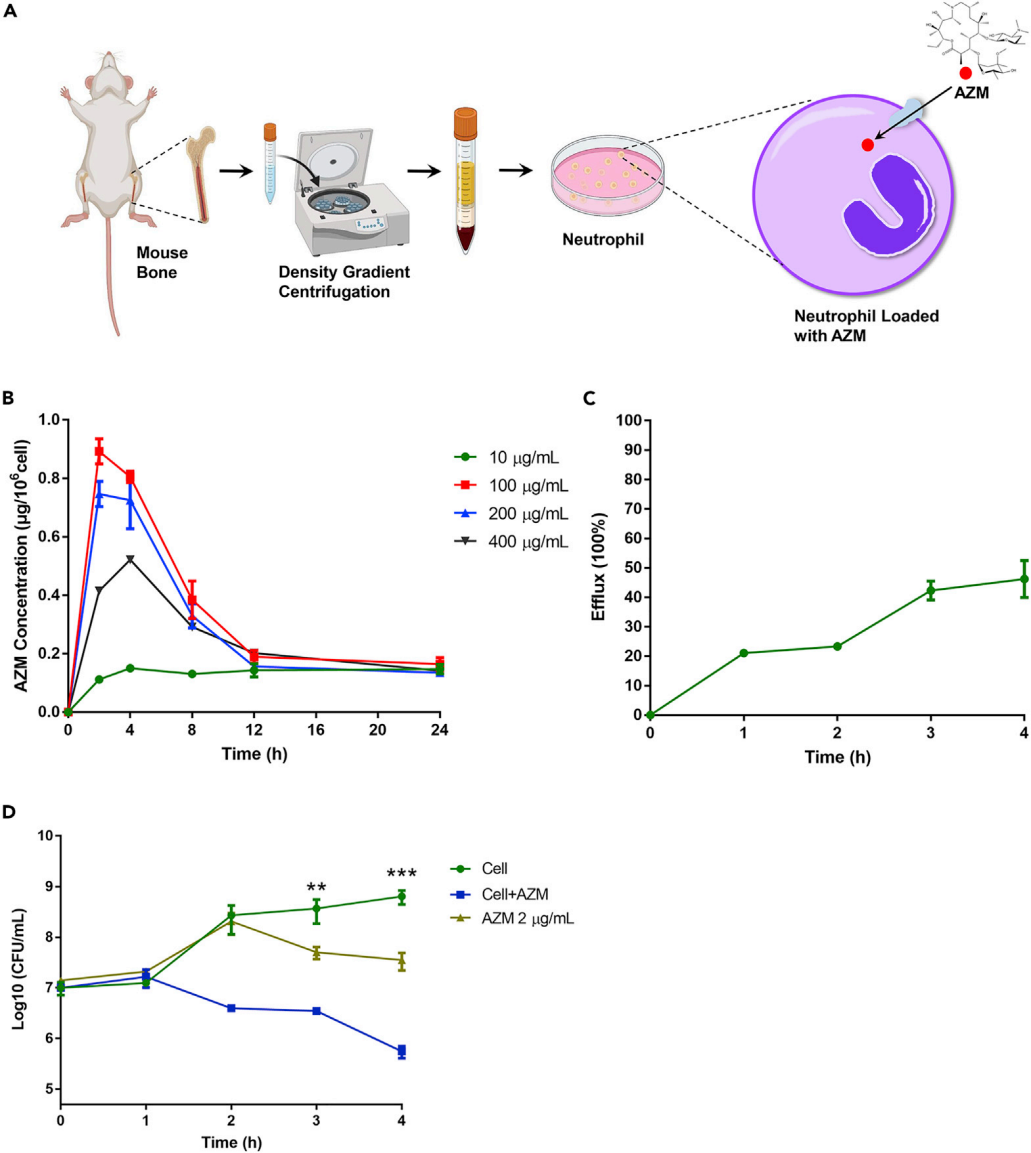
Uptake of azithromycin by neutrophils and the bacteria-killing efficacies of the neutrophils (A) Schematic illustration of the isolation of neutrophils and uptake of azithromycin by neutrophils. (B) Uptake kinetics of azithromycin by neutrophils. The intracellular amounts of azithromycin were determined by ELISA. Results represent means ± SD. (C) Release of azithromycin after neutrophils were incubated with 100 μg/mL azithromycin for 2 h. The extracellular concentrations of azithromycin were determined by ELISA. Results represent means ± SD. (D) Time-kill curves of neutrophils against PA14. Neutrophils were incubated in RPMI with 20% FBS in the absence or presence of 100 μg/mL azithromycin for 2 h. After resuspension with RPMI, the neutrophils at the concentration of 2×10^6^ cells/mL were incubated with 2 × 10^7^ CFU/mL PA14 (total volume = 1 mL). Meanwhile, the same amount of PA14 cells was incubated with or without 2 μg/mL azithromycin in RPMI. At indicated time points, the live bacteria numbers were determined by plating. Results represent means ± SD, ∗∗, p < 0.01; ∗∗∗, p < 0.001 by Student’s *t* test.

Enrichment of azithromycin in human neutrophils has been observed *in vitro* and in human bodies (Wilms et al., 2006; Hand and Hand, 2001). In the presence of azithromycin at concentrations equal to or lower than the human peak plasma drug concentration (1 μg/mL), the cellular/extracellular concentration ratio of azithromycin reached more than 100 in neutrophils (Vazifeh et al., 1997), indicating that neutrophils are natural delivery vectors of azithromycin. We hypothesized that incubation with higher concentrations of azithromycin might increase the intracellular drug concentration and subsequently enhance the bactericidal efficacy of the cells. We thus isolated neutrophils from mouse bone marrow with a purity of approximately 90% (Figure S1A) and Diff-Quik staining showed the typical lobular shape of the neutrophil nuclei (Figure S1B). The neutrophils were incubated with azithromycin at concentrations ranging from 10 to 400 μg/mL (Figure 2A). The highest intracellular drug accumulation was observed after incubation with 100 μg/mL azithromycin for 2 h (Figure 2B). Higher concentrations of azithromycin or prolonged incubation reduced the intracellular drug levels, which might be owing to the immunomodulatory or toxic effect of azithromycin.

We then determined the drug efflux after the cells were incubated with 100 μg/mL azithromycin for 2 h. Approximately 80% and 60% of the intracellular azithromycin were retained after 2 and 4 h, respectively (Figure 2C). Incubation with azithromycin increased the bactericidal ability of the neutrophils against a wild-type *P. aeruginosa* reference strain PA14 (Figure 2D).

### Development of neutrophils loaded with colistin and azithromycin

**Figure 3:**
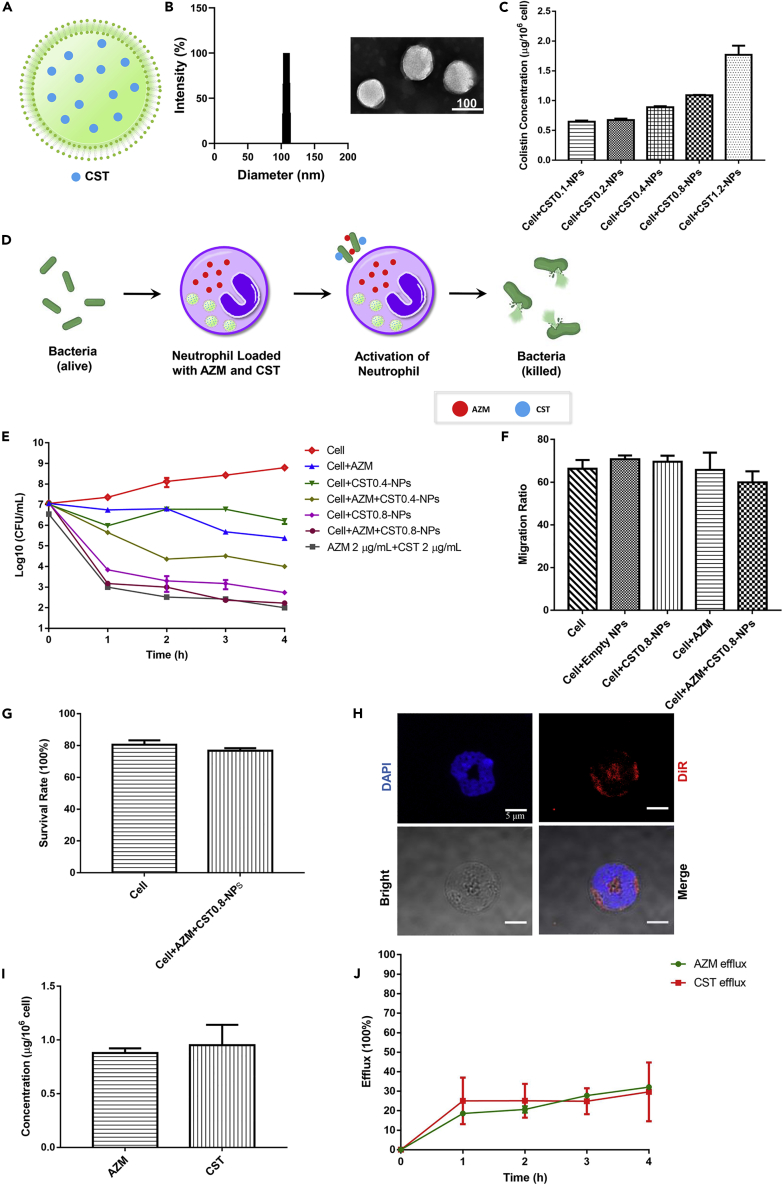
Uptake of colistin by neutrophils and the effects of intracellular azithromycin and colistin on bactericidal efficacies and migration of neutrophils (A and B) (A) Schematic illustration of CST-liposomes (B) Particle sizes of the colistin-containing liposomes (CST-NPs) were measured by dynamic light scatting (DLS) and observed by transmission electron microscope (TEM) images. (C) Uptake of colistin by mouse neutrophils. Neutrophils were incubated with indicated amounts of the CST-NPs for 2 h. The intracellular amounts of colistin were determined by ELISA. Results represent means ± SD. (D) Schematic illustration of the bacteria-killing experiment with neutrophils loaded with azithromycin and colistin. (E) 2×10^6^ cells/mL neutrophils loaded with azithromycin and colistin alone or in combination were incubated with 2 × 10^7^ CFU/mL PA14. Meanwhile, the same amount of bacterial cells was incubated with 2 μg/mL azithromycin and 2 μg/mL colistin in RPMI. At indicated time points, the live bacteria numbers were determined by plating. Results represent means ± SD. (F) The migration abilities of the neutrophils were determined using a transwell assay. Results represent means ± SD. (G) Survival rates of neutrophils 2 h after incubation with AZM and CST0.8-NPs. Results represent means ± SD. (H) Representative imaging of neutrophils loaded with DiR-labeled liposomes. The nuclei of neutrophils were stained with DAPI and the liposomes were labeled with DiR. (I) Neutrophils were incubated with 100 μg/mL azithromycin and CST0.8-NPs for 2 h. The intracellular amounts of azithromycin and colistin were determined by ELISA. Results represent means ± SD. (J) Release of azithromycin and colistin from the neutrophils was determined by ELISA at indicated time points after the incubation. AZM, azithromycin; CST, colistin. Results represent means ± SD.

Colistin and azithromycin have been shown to synergistically kill Gram-negative bacteria (Bae et al., 2016). Therefore, simultaneous loading of the two drugs might further enhance the bactericidal ability of neutrophils. However, colistin is not directly taken up by neutrophils according to our observation, presumably owing to the large molecular size of colistin and lack of transportation system in the cell. To achieve the goal, we utilized a fusogenic liposome as a nanocarrier to encapsulate colistin (designated as CST-NPs) and deliver the drug into neutrophils. The fusogenic liposome was composed of 1, 2-dimyristoyl-*sn*-lycero-3-phosphocholine (DMPC), 1, 2-distearoyl-*sn*-glycero-3-phosphoethanolamine-N [methoxy (polyethylene glycol)-2000] (DSPE-PEG2000), and 1, 2-dioleoyl-3-trimethylammonium-propane (DOTAP). The fusogenic feature of the liposome is endowed by a controlled ratio of structure, cationic and PEGylated lipid components, as evidenced by a previous report (Kim et al., 2018). In a typical procedure, the fusogenic liposomes were prepared using the established film hydration method, followed by extrusion through a 100 nm polycarbonate membrane. During the hydration of lipid components, the colistin was loaded into the inner hydrophilic cavity of the fusogenic liposome (Figure 3A). The size distribution and morphology of the CST-NPs were shown in Figure 3B. The diameters of the CST-NPs were around 110 nm and the colistin loading efficiency of the liposomes was around 40 mg/mL.

Neutrophils were incubated with the CST-NPs at various ratios, ranging from the colistin amounts equivalent to 0.1 mg/mL to 1.2 mg/mL (designated as CST0.1-NPs and CST1.2-NPs) for 2 h. Following incubation, the extracellular liposomes were removed by centrifugation and washing with PBS. The intracellular accumulation of colistin increased in a dose-dependent manner (Figure 3C). However, CST1.2-NPs resulted in 51.76% lysis of the cells, indicating a cytotoxic effect. CST0.8-NPs and CST0.4-NPs resulted in 25.88% and 11.76% lysis, respectively. We then compared the bactericidal activities of the cells incubated with CST0.4-NPs and CST0.8-NPs liposome in the absence and presence of 100 μg/mL azithromycin (Figure 3D). Neutrophils incubated with CST0.4-NPs and CST0.8-NPs resulted in approximately 10- and 2 × 10^4^-fold killing of PA14 after 4 h, which were further enhanced by approximately 100- and 5-fold by the coincubation with azithromycin, respectively (Figure 3E), indicating a stronger bactericidal effect by the neutrophils incubated with azithromycin and CST0.8-NPs.

As the neutrophil-mediated drug delivery requires efficient recruitment of the neutrophils to the infected tissue, we determined whether the liposome and azithromycin affect the migration ability and viability of the cells. As shown in Figure 3F, incubation with CST0.8-NPs in the absence and presence of azithromycin did not affect the cell migration in a transwell assay. Meanwhile, the survival rate of the drug-loaded neutrophils was similar to that of the drug-free cells 2 h after incubation with azithromycin and CST0.8-NPs (Figure 3G).

Based on the effects of the liposome and azithromycin on the cell viability, bactericidal and migration abilities, we chose CST0.8-NPs (20 μL of the liposome), 100 μg/mL azithromycin, and 2-h incubation as the condition for our following experiments. By using 1,1′-dioctadecyl-3,3,3′,3′-tetramethylindotricarbocyanine iodide (DiR) labeled liposomes, we found that the liposomes were localized in the cytoplasm of the neutrophil (Figure 3H). The intracellular amounts of azithromycin and colistin were 0.88 ± 0.05 μg and 0.95 ± 0.18 μg per 10^6^ cells, respectively (Figure 3I), which were relatively stable in our following experiments. Based on the amounts of the extracellular and intracellular colistin amounts, the uptake efficacy is 0.125%. As stable intracellular retaining of the drugs before encountering bacteria is essential for the treatment effectiveness, we monitored the intracellular drug amounts. 4 h after incubation with CST0.8-NPs and azithromycin, about 70% of each of the drugs remained in the cells (Figure 3J).

**Figure 4:**
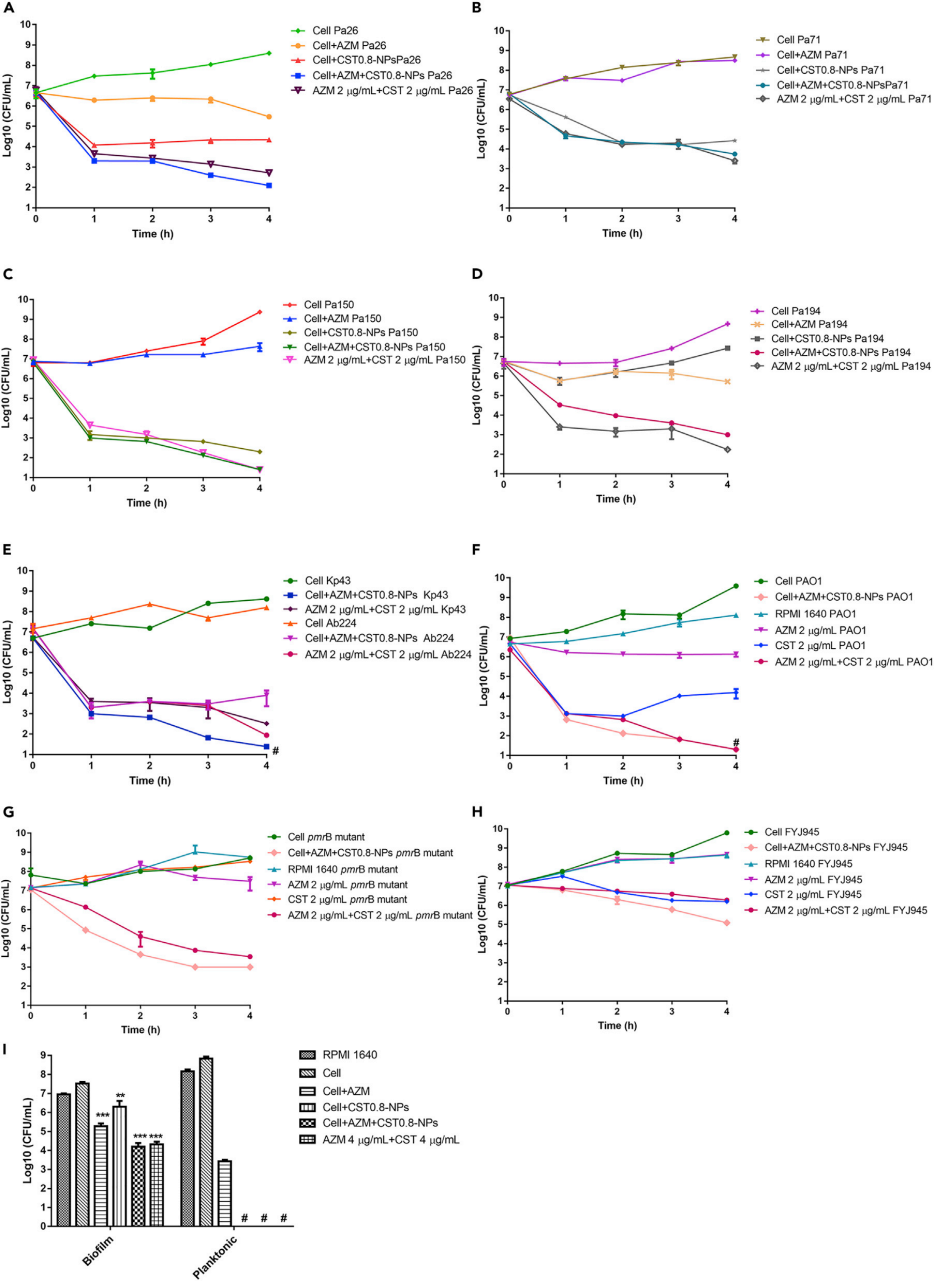
Killing efficacies of neutrophils loaded with azithromycin and colistin (A–E) Neutrophils at the concentration of 2×10^6^ cells/mL were incubated with indicated strains at 2 × 10^7^ CFU/mL (neutrophils:bacteria = 1:10). Meanwhile, the same amount of bacterial cells was incubated with 2 μg/mL azithromycin and 2 μg/mL colistin individually or in combination in RPMI. (a-e) Time-kill curves of neutrophils loaded with azithromycin and colistin against 4 clinical isolates of *P*. *aeruginosa* Pa26 (A), Pa71 (B), Pa150 (C), Pa194 (D), a *K. pneumoniae,* and an *A. baumannii* clinical isolates (Kp43 and Ab224) (E). Results represent means ± SD. (F–H) Time-kill curves of azithromycin, colistin, or neutrophils loaded with azithromycin and colistin against wild-type PAO1 (F), the *pmrB* mutant (G), and the *K. pneumoniae* strain FYJ945 (H). Results represent means ± SD. (I) Killing efficacies of neutrophils loaded with azithromycin and colistin against the biofilm of PA14. Results represent means ± SD. ∗∗, p < 0.01, ∗∗∗, p < 0.001 compared to the RPMI control by Student’s *t* test. #, < limit of detection (25 CFU/mL). AZM, azithromycin; CST, colistin.

We next examined the killing efficacies against clinical Gram-negative pathogens. The drug sensitivity (MIC) profile of each isolate was shown in Table S1. The drug-loaded neutrophils exhibited efficient bactericidal activities against six carbapenem resistance clinical isolates, including four *P. aeruginosa* strains, an *A. baumannii* strain, and a *K. pneumoniae* strain (Figures 4A–4E).

In the clinic, bacteria develop resistance to colistin owing to the mutation or acquisition of resistant genes, which might leave the infection incurable. To examine the killing efficacies of the drug-loaded neutrophils against colistin-resistant strains, we utilized a *pmrB* mutant derived from a wild-type *P. aeruginosa* reference strain PAO1 in an evolution experiment and a *mcr-1* containing *K. pneumoniae* strain (FYJ945). Mutation of *pmrB* results in the upregulation of the *arnB* operon, which adds 4-amino-*l*-arabinose addition lipid A (Jeddou et al., 2020). Mcr-1 adds a phosphoethanolamine (PEA) group to lipid A (Xu et al., 2018). Both of the modifications reduced the affinity between LPS and colistin, thus increasing bacterial resistance (Jeddou et al., 2020; Xu et al., 2018). The MICs of colistin for PAO1, the *pmrB* mutant, and FYJ-945 were 0.0625, 32, and 16 μg/mL, respectively (Table S1). The bacteria were treated with neutrophils loaded with azithromycin and colistin or the drugs at amounts equal to those carried by the neutrophils individually or in combination. The drug-loaded neutrophils resulted in >10^6^ and 10^4^-folds killing the wild-type PAO1 and the *pmrB* mutant, respectively (Figures 4F and 4G). The difference might be owing to the higher colistin tolerance of the *pmrB* mutant (Figure 4G). The *K. pneumoniae* strain FYJ945 was highly tolerant to both azithromycin and colistin (Table S1). Azithromycin alone did not inhibit bacterial growth. Colistin alone or in combination with azithromycin reduced the bacterial number by approximately 5-folds. However, the drug-loaded neutrophils resulted in 100-folds of bacterial killing (Figure 4H).

In the treatment of chronic infections, it is critical to promote the biofilm clearance ability of neutrophils. As shown in Figure 4I, neutrophils alone were unable to reduce the live bacterial number in biofilm. However, neutrophils loaded with either azithromycin or colistin reduced the biofilm-associated bacteria number by 40- or 8-fold, and cells containing both drugs reduced the bacteria number by 1000-fold. In combination, these results demonstrated an effective bactericidal activity of the drug-loaded neutrophils.

### Responses of the drug-loaded neutrophils to *P. aeruginosa*

**Figure 5:**
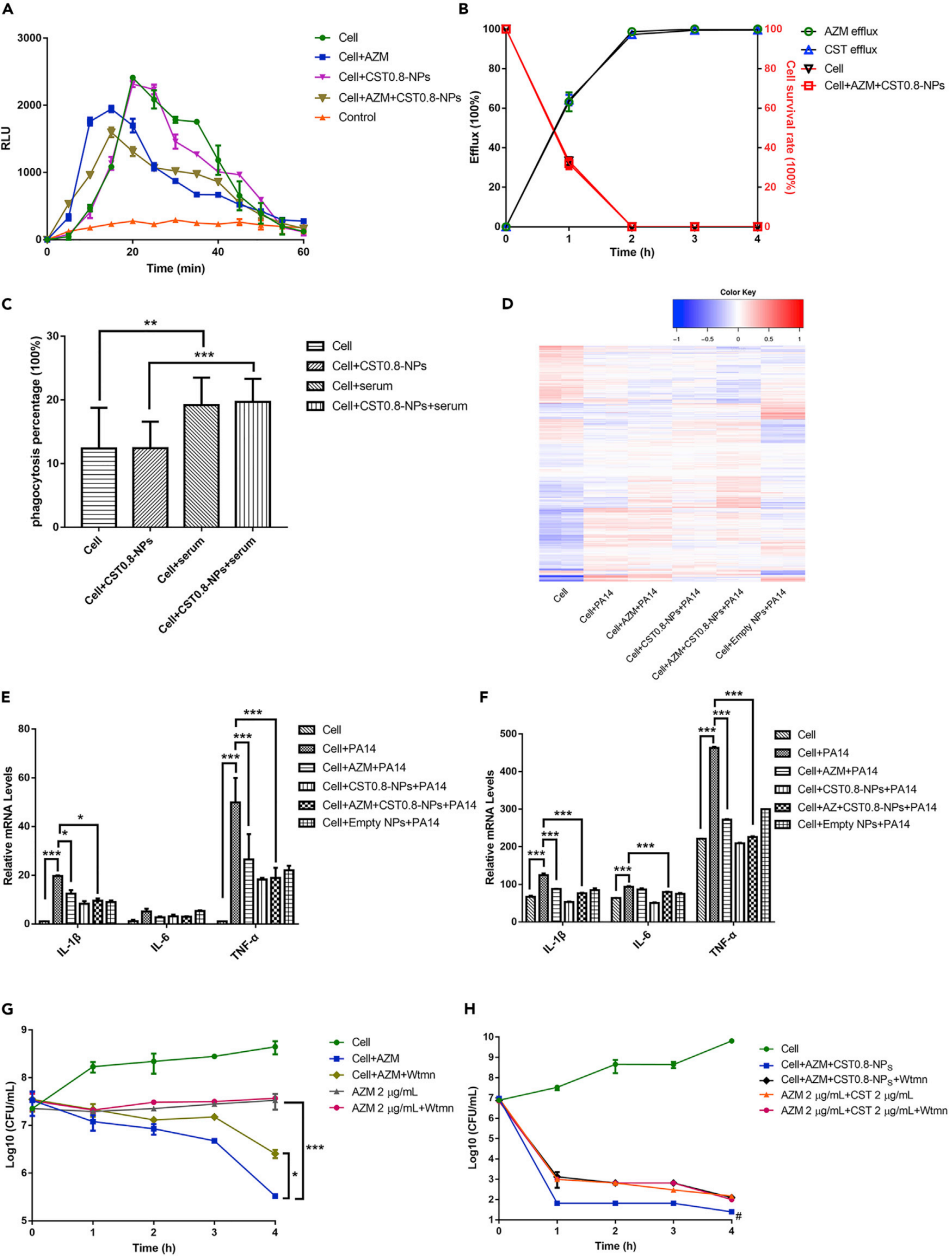
Effects of the intracellular azithromycin and colistin on neutrophil’s responses to bacteria (A) ROS production by neutrophils. Neutrophils loaded with azithromycin, colistin, or both drugs were incubated with PA14 (neutrophils:bacteria = 1:10). The production of ROS was monitored every 5 min for 1 h. Results represent means ± SD. (B) Release of colistin and azithromycin from drug-loaded neutrophils in the presence of PA14. The extracellular drug concentrations were determined by ELISA. The cell survival percentages were determined by trypan blue staining. Results represent means ± SD. (C) Fluorescent bacteria (PA14/pUCP20-*gfp*) were incubated with drug-loaded and drug-free neutrophils with or without the anti-PA14 serum (1:10 dilution) for 10 min. Phagocytosis of the bacteria was observed by confocal microscopy. Results represent means ± SD. (D) Clustered heatmap of differently expressed genes by RNA-seq. (E and F) Relative mRNA levels (E) and secretion (F) of the cytokine genes in neutrophils with or without drug loading after incubation with PA14 at a ratio of 10:1 for 2 h ∗, p < 0.05, ∗∗∗, p < 0.001 by two-way ANOVA. Results represent means ± SD. (G and H) Time-kill curves of drug-loaded cells against PA14 (G) and Kp43 (H) in the presence and absence of 100 nM wortmannin. #, < limit of detection (25 CFU/mL). Wtmn, wortmannin. AZM, azithromycin; CST, colistin. Results represent means ± SD.

To examine the effects of the loaded drugs on the neutrophils’ responses to bacteria, neutrophils were incubated with 100 μg/mL azithromycin and CST0.8-NPs for 2 h, Diff-Quik staining confirmed there was no morphological difference between neutrophils and neutrophil loaded azithromycin and colistin (Figure S1C). We then monitored ROS generation. The cells were incubated with the PA14 cells at a ratio of 1:10. The ROS generated by the drug-free neutrophils peaked at 20 min after encountering the bacteria (Figure 5A). Loading with colistin did not affect the ROS generation, however, azithromycin slightly quickened the response and reduced the maximal ROS level (Figure 5A). Nevertheless, the ROS generated by the neutrophils with or without the drugs diminished 1 h after encountering the bacteria (Figure 5A), presumably owing to cell death. Indeed, incubation with the bacteria resulted in approximately 70% and 100% death of the neutrophils after 1 and 2 h, respectively (Figure 5B). Meanwhile, the release of the loaded drugs corresponded to cell death (Figure 5B). In addition, the drug-free and drug-loaded neutrophils displayed similar phagocytosis abilities in the presence and absence of the antiserum against PA14 (Figures 5C and S2).

### Interplay between neutrophils and the loaded drugs in response to bacteria

To examine the effects of the loaded drugs on the neutrophils’ response to *P. aeruginosa*, we reduced the cells to bacteria ratio to 0.1, which resulted in around 83% survival of the cells 2 h after the incubation (data not shown). Transcriptomic profiles of the cells were analyzed by RNA-seq. Compared to the drug-free neutrophils, 127 and 202 genes were up- and down-regulated in the drug-loaded neutrophils (Table S2). As previous studies demonstrated that the reduction of inflammation resulted in better therapeutic outcomes in *P. aeruginosa* lung infections (Cohen and Prince, 2013; Véliz Rodriguez et al., 2012; Wonnenberg et al., 2016), we focused on the proinflammatory cytokine genes. Consistent with previous reports, azithromycin reduced the expression of genes encoding IL-1β, IL-6, and TNF-α with or without colistin (Figure 5D and Table S2) (Cigana et al., 2006; Meyer et al., 2009). Interestingly, the empty liposome reduced the expression of the proinflammatory genes for an unknown reason (Figure 5D and Table S2). qRT-PCR and ELISA confirmed the downregulation of IL-1β, IL-6, and TNF-α genes and reduced the production of the cytokines by azithromycin and the liposome (Figures 5E and 5F), indicating immunomodulatory effects on the neutrophils.

Our results demonstrated a stronger killing effect on PA14 by the azithromycin carrying neutrophils than the equal amount of azithromycin (Figure 2D). Previous studies demonstrated synergistic bactericidal effects between ROS and antibiotics (Mandal et al., 2019). To examine whether the neutrophil-generated ROS contributes to the enhanced bacterial killing, we utilized wortmannin to inhibit ROS generation by neutrophils (Sue-A-Quan et al., 1997). Compared to azithromycin carrying neutrophils, the presence of wortmannin increased the survival of PA14 (Figure 5G). As for the combination of azithromycin and colistin, treatment with the antibiotics and drug-loaded neutrophils resulted in similar killing effects on PA14, which might be owing to the high susceptibility of PA14 to the antibiotic combination (Figure 3E). We thus used the *K. pneumoniae* clinical isolate Kp43, against which the drug-loaded neutrophils displayed a stronger killing effect than the antibiotic combination (Figure 4E). Treatment with wortmannin reduced the bactericidal effect of the drug-loaded neutrophils to a similar level as the antibiotic combination (Figure 5H). Collectively, these results demonstrate that the neutrophil-generated ROS might enhance the bactericidal effect of the antibiotics.

### *In vivo* therapeutic efficacy of the colistin and azithromycin-loaded neutrophils

**Figure 6:**
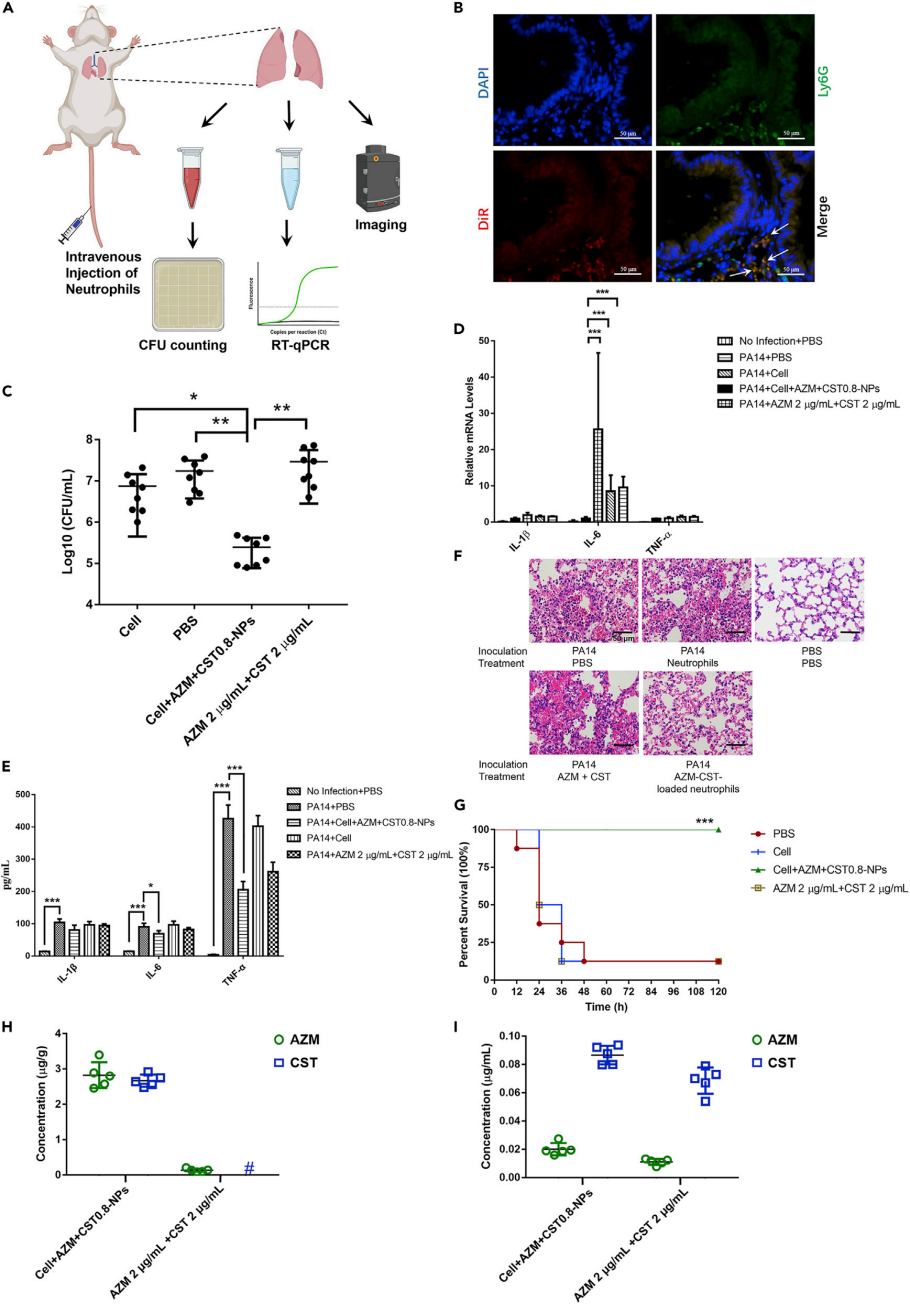
Therapeutic effects of drugs loaded neutrophils against *P*. *aeruginosa* infection in the acute pneumonia model (A) Schematic illustration of the mouse acute pneumonia model. Each mouse was intranasally inoculated with 4 × 10^6^ CFU of PA14. (B) Representative images showing DiR-labeled neutrophils in a frozen section. 2 h postinfection (hpi), each mouse was injected intravenously with neutrophils that had been incubated with DiR-labeled liposomes. 2 h afterward, the lungs were isolated and fixed. The cell nuclei were stained with DAPI. Neutrophils were stained with a Ly6G antibody. The white arrows in the merged panel indicate the fluorescent-labeled neutrophils in the alveolar space. (C–E). 2 h postinfection (hpi), each mouse was injected intravenously with PBS, neutrophils without drug, neutrophils loaded with azithromycin and colistin, or azithromycin and colistin at the dosages equivalent to those carried by the neutrophils (8 mice per group). (C) The bacterial loads in the lungs were determined at 10 hpi. The central bar indicates the mean, and the error bar indicates standard error. (D) At 10 hpi, the lungs were isolated. The relative mRNA levels of genes encoding IL-1β, IL-6 and TNF-α were determined by real-time PCR. Results represent means ± SD. ∗, p < 0.05, ∗∗, p < 0.01, ∗∗∗, p < 0.001 by two-way ANOVA. (E) At 10 hpi, the lungs were isolated and homogenized. The IL-1β, IL-6, and TNF-α levels in the supernatants were determined by ELISA. Results represent means ± SD. ∗, p < 0.05, ∗∗∗, p < 0.001 by two-way ANOVA. (F) Hematoxylin and eosin (H&E) staining of the mice lungs. (G) The survival of the infected mice was monitored for 5 days ∗∗∗, p < 0.001 compared to the other groups by a log rank test with Prism software. (H) At 4 hpi, the lungs were isolated and homogenized, the drug concentrations were determined by ELISA. #, < limit of detection (0.5 μg/g). The central bar indicates the mean, and the error bar indicates standard error. (I) At 4 hpi, the drug concentrations in the sera were determined by ELISA. The central bar indicates the mean, and the error bar indicates standard error.

We next utilized a murine acute pneumonia model to evaluate the treatment efficacy of the neutrophil-mediated drug delivery system (Figure 6A). Mice were infected intranasally with wild-type PA14. 2 h postinfection, 2×10^6^ neutrophils with or without incubation with 100 μg/mL azithromycin and CST0.8-NPs were injected into the tail veins of the mice. Based on the amounts of the neutrophil-carried antibiotics, each mouse received approximately 2 μg azithromycin and 2 μg colistin. By using fluorescence-labeled liposome, we found that the injected neutrophils were observed in the infected lungs (Figures 6B and S3A), which is consistent with previous reports (Chu et al., 2015; Fan et al., 2021). Meanwhile, the fluorescence was observed in the livers and spleens, whereas the signals in the hearts and kidneys were minimal (Figure S3A). Previous studies in mice demonstrated that pneumonia in the lung induces inflammatory response in liver (Odom et al., 2021; Quinton et al., 2012). In addition, acute *P. aeruginosa* lung infections result in bacterial dissemination in blood and liver (Pène et al., 2012; Gonçalves-de-Albuquerque et al., 2016; Ren et al., 2019). We thus suspected that the recruitment of the neutrophils to the liver and spleen might be owing to inflammatory responses and the presence of bacteria. Indeed, we found higher bacterial loads and stronger inflammatory responses in the livers and spleens than those in the hearts and kidneys (Figures S3A and S3B), which might explain the recruitment of neutrophils.

Compared to PBS or neutrophils alone, a single injection with the drug-loaded neutrophils significantly reduced the bacterial loads and reduced the expression levels and secretion of IL-1β, IL-6, and TNF-α in the lungs 10 h postinfection (Figures 6C-6E). Hematoxylin and Eosin (H&E) staining further verified the alleviation of inflammation by the drug-loaded neutrophils (Figure 6F). In addition, the injection of the drug-loaded neutrophils resulted in a 100% survival rate of the infected mice, whereas the same number of drug-free neutrophils exhibited no protective effect (Figure 6G). We then examined the therapeutic effect of direct injection of the two antibiotics. Injection with the same amounts of the antibiotics (2 μg azithromycin and 2 μg colistin per mouse) neither reduced the bacterial load nor conferred protection (Figures 6C and 6G).

To compare the drug delivery efficacy by the neutrophils with direct injection, we measured the drug concentrations in the lungs and blood. In the infected lungs, injection of the drug-loaded neutrophils resulted in 2.82 ± 0.36 μg/g azithromycin and 2.67 ± 0.17 μg/g colistin, whereas injection of free drugs resulted in 0.13 ± 0.05 μg/g azithromycin and colistin below our detection limit (Figure 6H). Meanwhile, injection of the drug-loaded neutrophils resulted in lower blood concentrations of azithromycin (0.01 ± 0.002 μg/mL) and colistin (0.06 ± 0.009 μg/mL) than those in the mice received free drug injection (0.02 ± 0.004 μg/mL azithromycin and 0.08 ± 0.006 μg/mL colistin) (Figure 6I). These results demonstrated targeted delivery of the antibiotics by the neutrophils.

It is worth mentioning that the doses of azithromycin and colistin for humans are 10 mg/kg and 2.5 mg/kg, respectively (Viscardi et al., 2013; Nation et al., 2016). According to the Meeh-Rubner equation (Spiers and Candas, 1984), the corresponding azithromycin and colistin doses in mice are approximately 91 mg/kg and 22.75 mg/kg. Thus, our neutrophil-mediated drug delivery system achieved effective protection with azithromycin and colistin at 0.1 and 0.4% of the normal respective doses.

To evaluate the safety of the neutrophil-mediated drug delivery, we injected the drug-loaded neutrophils or PBS into uninfected mice. The weight gain of the mice that received the neutrophils was similar to those that received PBS in the following 14 days (Figure S4A). On the last day, the mice were sacrificed. H&E staining revealed normal histology of the organs, including lung, heart, liver, spleen, and kidney (Figure S4B). In combination, no obvious toxicity was observed for the drug-loaded neutrophils.

To examine whether our method is applicable to human neutrophils, we isolated neutrophils from human blood and incubated the cells with azithromycin and the CST-liposome as performed on the mouse neutrophils. The drug-loaded neutrophils exhibited efficient killing activities against the strains of PA14, Pa150, Kp43, and Ab224 (Figure S5). Collectively, these results demonstrated that loading with azithromycin and colistin enhanced the bactericidal abilities of neutrophils and modulate the inflammatory responses of the cells.

## Discussion

The current development of new antibiotics is way slower than the bacterial development of resistance. Therefore, effective treatment strategies with currently available antibiotics are one of the options in dealing with the antimicrobial resistance crisis. Features of the ideal treatment strategy include targeted drug delivery, efficient killing of pathogens, and appropriate inflammatory responses. In this study, we developed super-neutrophils by loading the cells with azithromycin and colistin. By utilizing a murine acute pneumonia model, we demonstrated that neutrophils carrying the drugs effectively protected mice in a *P. aeruginosa* pneumonia model.

In response to inflammatory stimuli, neutrophils are rapidly recruited to the inflammatory sites through extravasation, making them an efficient drug delivery system to treat infection, tumor, and inflammatory disorders (Chu et al., 2018; Aitken et al., 2018; Dong et al., 2017, 2018; Chellappan et al., 2020). Granulocytes transfusion is considered a potential therapeutic strategy to treat severe infectious diseases in patients with neutropenia or functional neutrophil disorders (West and Conry-Cantilena, 2019). The main complications of granulocyte transfusion include human leukocyte antigen sensitization, fever, pulmonary reactions, and alloimmunization (West and Conry-Cantilena, 2019; Gea-Banacloche, 2017). In this study, we found no significant difference in the body weight between the mice that received the drug-loaded neutrophils with those that received PBS. In addition, H&E staining revealed no obvious influence on the major organs, including heart, liver, lung, kidney, and spleen. Further comprehensive evaluation of the safety of the drug-loaded neutrophils is warranted for this therapeutic strategy.

Choices of drugs carried by neutrophils are critical for the success of the treatment. In this study, we chose azithromycin and colistin to treat bacterial infections by considering the following aspects: (1) intracellular accumulation of the drugs; (2) antimicrobial synergy between the drugs; (3) influences on the functions of neutrophils.

Azithromycin is a macrolide antibiotic that has been shown to reach the highest cellular/extracellular concentration ratio (>100) in neutrophils compared to other macrolides and antibiotics in different categories, including β-lactams, aminoglycosides, quinolones, tetracyclines, sulfonamides, and so forth (Vazifeh et al., 1997). The cellular sodium/calcium exchanger and nucleoside transporter are involved in the energy-requiring uptake of azithromycin (Dong et al., 2018; West and Conry-Cantilena, 2019). Inside neutrophils, azithromycin is accumulated in granules or bind to unknown cytoplasmic proteins, which might contribute to the stable retaining of the drug (Laufen et al., 1990; Hand et al., 1983). Our results demonstrated that *ex vivo* incubation with azithromycin at 100 μg/mL for 2 h resulted in an intracellular accumulation of 1 μg/10^6^ cells and around 75% of the drug retained in the cell 2 h after removal of the extracellular azithromycin.

At subinhibitory concentrations, azithromycin exhibits antivirulence activity mainly through repressing the expression of quorum sensing system-regulated virulence factors in *P.* *aeruginosa* (Gillis and Iglewski, 2004; Favre-Bonté et al., 2003; Tateda et al., 2001; Molinari et al., 1992, 1993). Azithromycin binds to the 50S subunit of ribosome and causes ribosome stalling, resulting in the depletion of the intracellular tRNA pool, which might repress the translation of mRNAs with rare codons to a greater extent (Gödeke et al., 2013). The quorum sensing regulatory gene *rhlR* is more vulnerable to the translational repression by azithromycin owing to its rare codon (Gödeke et al., 2013), thus leading to the repression of quorum sensing regulated virulence factors and subsequent inhibition of biofilm formation or disruption of biofilm. A recent study revealed that azithromycin induces changes in the 3′ end formation of mRNAs in *P. aeruginosa*, which results in repression of genes related to quorum sensing, biofilm formation, and stress responses (Konikkat et al., 2021).

Colistin and polymyxin B are cyclic polypeptide antibiotics that are composed of a fatty acid chain, a linear tripeptide, and a cyclic heptapeptide (Soon et al., 2011). Based on the amounts of the extracellular and intracellular colistin amounts, the uptake efficacy is 0.125%. Longer incubation increased colistin uptake; however, the cytotoxicity was also increased, presumably caused by the liposome components. Further studies are warranted to reduce the cytotoxicity and increase the uptake efficacy. Fluorescence microscopy demonstrated cytoplasmic localization of the colistin-containing liposomes in the neutrophils (Figure 3H). Previous studies also demonstrated phagocytosis and cytoplasmic localization of liposomes in neutrophils (Che et al., 2020; Han et al., 2021). Therefore, colistin might exist in the liposomes inside the neutrophils.

It has been demonstrated that antimicrobial peptides and colistin-induced damages to the bacteria outer and inner membrane facilitate the intracellular accumulation of azithromycin, thus achieving synergy (Lin et al., 2015; Velkov et al., 2013, 2014; Krzyzanski and Rao, 2017; Belanger et al., 2020). Consistent with the previous report, we found that the combination of colistin with azithromycin resulted in superior bactericidal effects to those of the individual drugs. It has been demonstrated that ROS facilitates the killing of bacteria by antibiotics and elimination of bacterial persister cells (Dubey et al., 2021). Upon encountering bacteria, neutrophils promptly generate ROS (Zeng et al., 2019). Meanwhile, the intracellular azithromycin and colistin might be released by degranulation. By utilizing wortmannin to block ROS generation, we found that the neutrophil-generated ROS facilitates the bacterial killing by azithromycin and colistin (Figures 5G and 5H), which might be owing to the ROS caused damage to bacterial membrane, protein, and DNA (Zeng et al., 2019). However, in the presence of wortmannin, the killing efficacy of the azithromycin carrying neutrophils was still higher than azithromycin alone (Figure 5G), which might be attributed to the neutrophil-released antimicrobial peptides (Lin et al., 2015).

Besides antimicrobial effects, azithromycin has been demonstrated to exhibit immunomodulatory effects in humans by regulating the NF-κB signaling pathway, inflammasome activation, and autophagy flux (Venditto et al., 2021). It has been widely used in the treatment of bacterial infections in the upper respiratory tract (Davidson, 2019). Consistent with previous reports, we found the downregulation of multiple inflammatory cytokine genes in the drug-loaded neutrophils, which might contribute to the alleviation of tissue damage. In addition, genes involved in signaling transduction and inflammatory response were downregulated in the drug-loaded neutrophils, such as *PTGS2*, *GBP2*, and *CLEC4E* (Table S2). *PTGS2* encodes prostaglandin-endoperoxide synthase 2, which generates prostaglandins that play important roles in inflammation (Hellmann et al., 2015). *GBP2* encodes guanylate binding protein 2 that promotes the activation of caspase-11 inflammasome and pyroptosis (Kopitar-Jeral, 2017; Meunier et al., 2014; Pilla et al., 2014). *CLEC4E* encodes the C-type lectin receptor Mincle that recognizes glycolipids from pathogens and endogenous lipid-based damage-associated molecular patterns, leading to the activation of inflammatory responses (Lu et al., 2018). We also found that the liposome reduced inflammatory response. The RNA-seq results revealed the downregulation of the *MYD88* and *NLRP3* genes (Table S2) by the liposome, which might reduce the Toll-like receptor and inflammasome-mediated responses to bacterial ligands, respectively (Yambe et al., 2021; Hamerman et al., 2004; Kelley et al., 2019). Further studies are warranted to identify the intracellular target of azithromycin and the immunomodulatory mechanisms of the liposome.

Infection with PA14 resulted in severe occlusion with neutrophil infiltration and production of proinflammatory cytokines, which were reduced by injection with the drug-loaded neutrophils (Figures 6D–6F). Meanwhile, the bacterial loads in the lungs of mice were reduced by the drug-loaded neutrophils (Figure 6C). *In vitro* assays demonstrated that the loaded drugs significantly enhanced the bactericidal ability of the neutrophils (Figure 4), and azithromycin reduced the inflammatory response of the neutrophils (Figures 5D–5F). We thus suspected that the recruited drug-loaded neutrophils (Figure 6B) effectively reduced the bacterial load, which reduced the inflammatory response. In addition, the azithromycin carried by the neutrophils further alleviates the local inflammatory response. Although the alleviated inflammation reduced neutrophil recruitment, the infected mice were protected by the drug-loaded neutrophils (Figure 6G). A chronic infection model is warranted to further examine the neutrophil recruitment, inflammatory response, and bactericidal efficacy.

Besides recruitment to the infected lungs, the injected neutrophils were observed in the livers and spleens, which might be owing to the local inflammatory response and bacterial colonization (Figures S3A and 3B). The recruited neutrophils might contribute to the clearance of the bacteria and confer protection. In addition, the neutrophils might be isolated during isolation, which might result in homing to bone marrow and spleen (Swamydas and Lionakis, 2013). Thus, caution are needed to reduce neutrophil activation during *in vitro* isolation.

Overall, we demonstrated that neutrophils loaded with azithromycin and colistin exhibited efficient bactericidal and anti-inflammatory effects *in vitro* and *in vivo*. In addition, neutrophils might be used in the targeted delivery of effective antimicrobial agents that cannot be administered systemically owing to side effects or instability in blood, such as antimicrobial peptides.

### Limitations of the study

In this study, we focused on the protective effect of drug-loaded neutrophils in mice infected with *P. aeruginosa*. The drug-loaded neutrophils showed the effective bactericidal effect on other Gram-negative bacteria *in vitro*, whether the drug-loaded neutrophils can confer protection against other Gram-negative bacteria *in vivo* can be further researched. Meanwhile, real-time observation of the injected neutrophils might reveal the recruitment and distribution of the cells. Finally, it is worthy to examine the treatment efficacies in chronic infection models.

## STAR★Methods

### Key resources table

**Table:** 

REAGENT or RESOURCE	SOURCE	IDENTIFIER
**Antibodies**		

Azithromycin	Heowns	83,905-01-5
Colistin	Yuanye Bio	S17057-5g

**Bacterial and virus strains**		

*Pseudomonas aeruginosa* PA14	Laboratory Store	N/A
*Pseudomonas aeruginosa* Pa26	Clinical Isolate	N/A
*Pseudomonas aeruginosa* Pa71	Clinical Isolate	N/A
*Pseudomonas aeruginosa* Pa150	Clinical Isolate	N/A
*Pseudomonas aeruginosa* Pa194	Clinical Isolate	N/A
*Pseudomonas aeruginosa prmb* mutrant	PAO1 evolution	N/A
*klebsiella pneumoniae* Kp43	Clinical Isolate	N/A
*klebsiella pneumoniae* FYJ945	Zhejiang University	N/A
*Acinetobacter baumannii* Ab224	Clinical Isolate	N/A

**Chemicals, peptides, and recombinant proteins**		

PBS	Solarbio Life Sciences	PBS
Ethanol	Tianjin No.6 Chemical Reagent Factory	EtOH
1,2-dimyristoyl-*sn*-lycero-3-phosphocholine	Merck-Sigma-Aldrich	DMPC
1,2-distearoyl-*sn*-glycero-3-phosphoethanolamine-N [methoxy (polyethylene glycol)-2000]	Merck-Sigma-Aldrich	DSPE-PEG2000
1,2-dioleoyl-3-trimethylammonium-propane	Merck-Sigma-Aldrich	DOTAP

**Deposited data**		

Raw data	This study	NCBI BioProject: PRJNA869576
Software		
BioRender software	https://biorender.com	Schematic
Prime	GraphPad Software	Statistical

### Resource availability

#### Lead contact

Further information and requests for resources and reagents should be directed to and will be fulfilled by the lead contact Weihui Wu (wuweihui@nankai.edu.cn).

#### Materials availability

This study did not generate any new biological material.

### Experimental model and subject details

#### Animals

BALB/c female mouse (6-8 weeks, specific-pathogen-free) was purchased from Beijing Vital River Laboratory Animal Technology Co., Ltd. All animal experiments were performed in accordance with Chinese rules for the use of animals in research. All of the protocols were approved by the Animal Care and Use Committee of the College of Life Sciences, Nankai University (permission number NK-04-2012).

### Method details

#### Isolation of neutrophils

Mouse neutrophils were isolated from bone marrow by a mouse bone marrow neutrophil isolation kit (Solarbio, Beijing, China). Briefly, the mouse thigh bone was washed with 75% ethanol and phosphate buffered solution (PBS). The cells in the bone were flushed out by the RPMI 1640 medium with 20% fetal bovine serum (FBS), followed by centrifugation at 400*g* for 7 min and resuspended in PBS. Neutrophils were obtained by density gradient centrifugation as instructed by the manufacturer’s manual, and then washed twice with PBS. The purified neutrophils were cultured in RPMI 1640 medium with 20% FBS. The purity of the neutrophils was measured by flow cytometry analyses and Diff-Quik Stain (Leagene, Beijing, China).

Neutrophils from human peripheral blood were isolated by a human peripheral blood neutrophil separation kit (Solarbio, Beijing, China). Briefly, blood from healthy human volunteers was placed on top of the density gradient centrifugation reagent and centrifuged as instructed by the manufacturer’s manual. The cells were then washed three times with PBS. The purified neutrophils were cultured in RPMI 1640 medium with 20% FBS.

#### Uptake and release of azithromycin by neutrophils *in vitro*

To quickly determine the intracellular accumulation and efflux of the antibiotics by neutrophils under various conditions, we used ELISA to examine the antibiotic concentrations. The accumulation of azithromycin in neutrophils was examined by an ELISA kit (Jiangsu Wise Science and Technology Development, China). Purified neutrophils were incubated with 10–400 μg/mL azithromycin in the RPMI 1640 medium with 20% FBS. At indicated time points, the neutrophils were collected by centrifugation at 300*g* for 5 min and washed once with PBS. The cells were lysed by resuspension in 1 mL ddH_2_O, followed by centrifugation to remove the cell debris. The concentration of the azithromycin in the supernatant was determined by ELISA.

For the efflux essay, the purified neutrophils were incubated with 100 μg/mL azithromycin in the RPMI 1640 medium with 20% FBS. Two hours later, the cells were collected by centrifugation and resuspended in an azithromycin-free RPMI 1640 medium with or without PA14 (neutrophils:bacteria = 1:10). At indicated time points afterwards, the supernatant was collected after centrifugation and the azithromycin concentration was determined by ELISA.

#### Preparation of colistin loaded liposome

To obtain colistin loaded liposome (CST-liposome), the mixture of 1,2-dimyristoyl-*sn*-lycero-3-phosphocholine (DMPC), 1,2-distearoyl-*sn*-glycero-3-phosphoethanolamine-N [methoxy (polyethylene glycol)-2000] (DSPE-PEG2000), and 1,2-dioleoyl-3-trimethylammonium-propane (DOTAP) at a molar ratio of 76.2:3.8:20 was dissolved in anhydrous chloroform and then dried under a rotary evaporator. Afterwards, the dried lipid film was hydrated with aqueous solution of colistin (200 mg/mL), followed by extrusion through 400, 200 and 100 nm polycarbonate filters for 20 times. The resulting CST-liposome was purified by centrifugation at 4°C, followed by condensation with an Amico filter device with a molecule weight cutoff of 10 kDa (Millipore). Dynamic light scattering (DLS) was used to measure the hydrodynamic diameter of CST-liposomes (Malvern, Zetasizer).

#### Uptake and release of colistin by neutrophils *in vitro*

Neutrophils were incubated with the CST-liposome for 2 h in the RPMI 1640 medium with 20% FBS. To remove the extracellular liposome, the cells were washed twice with PBS. To determine the intracellular amount of colistin, the cells were lysed by resuspension in 1 mL ddH_2_O. The concentration of colistin in the supernatant was determined with an ELISA kit (Jiangsu Wise Science and Technology Development, China).

To monitor the release of colistin by neutrophils, the isolated neutrophils were incubated with CST-liposome and washed with PBS as aforementioned. Then the cells were incubated the RPMI 1640 medium containing 10% FBS with or without PA14 (neutrophils:bacteria = 1:10). The concentration of colistin in the supernatant was determined by ELISA.

#### Bacterial killing assay

Bacteria were grown to an OD_600_ of 1.0, then washed with PBS and resuspended in the RPMI 1640 medium. After incubation with azithromycin and CST-liposome individually or in combination, neutrophils were collected by centrifugation at 300*g* for 5 min, followed by washing with PBS twice. Then the neutrophils at the concentration of 10^6^ cell/mL were incubated with the bacteria at a ratio of 1:10 in 1 mL RPMI 1640 medium. At indicated time points (0–4 h), the number of live bacteria was determined by plating. When needed, 100 nM wortmannin was added to the neutrophils 5 min before incubation with the bacteria. To visualize the bacterial viability, 1 h after the incubation, the bacteria were collected by centrifugation at 8000*g* for 2 min, followed by staining with 10 μM propidium iodide (PI) in PBS at room temperature for 15 min. The bacteria were then observed by confocal microscopy.

For the biofilm killing assay, 1 × 10^6^ CFU of PA14 in 250 μL of RPMI 1640 medium were inoculated into each well of a 96-well plate. After incubation for 24 h at 37°C, the planktonic bacteria were removed and each well was gently washed twice with PBS. 250 μL of RPMI 1640 medium containing 10^6^ neutrophils and equal concentrations of the drugs (4 μg/mL azithromycin and 4 μg/mL colistin) was added to each well, followed by incubation for 4 h at 37°C with 5% CO_2_. The medium in each well was taken for the determination of live planktonic bacteria number. The attached bacteria were washed 3 times gently with PBS and dispersed by ultrasound as previously described (Ren et al., 2019). The live bacteria number was determined by plating.

#### Cell migration assay

The migration ability was determined by a transwell assay as previously described with minor modifications (Paulsson et al., 2010). Briefly, 10^5^ neutrophils with or without uptake of azithromycin and colistin were washed with PBS and resuspended in 100 μL RPMI 1640 with 0.1% FBS and then added into the upper well (3.0 μm pore size, 6.5 mm diameter inserts) of a 24-well plate. 600 μL RPMI 1640 with 0.1% FBS and 1 μΜ fMLP was added into the bottom well. After 2 h incubation at 37°C, the numbers of cells in the bottom well were counted with a hemocytometer.

#### ROS production by the neutrophils

ROS production by the neutrophils was measured as previously described with minor modifications (Wu et al., 2012). 1.0×10^6^ neutrophils were washed with PBS twice and resuspended in 200 μL PBS containing 100 μM luminol and 5 units of horseradish peroxidase, and added into each well of a 96-well plate. The cells were incubated at 37°C for 10 min, followed by incubation with the PA14 cells at a ratio of 1:10. The ROS production was monitored by measuring the relative light units (RLU) every 5 min for 1 h with a luminometer (Varioskan Flash; Thermo Scientific, USA).

#### Opsonophagocytic assays

The anti-PA14 serum was obtained by intranasal immunization of BALB/c mice twice with 1×10^6^ and 2 × 10^6^ CFU of PA14 at 1 week interval as previously described (Wu et al., 2012). 2 weeks after the last immunization, the serum was collected. GFP expressing PA14 (PA14/pUCP20-*gfp*) was grown to an OD_600_ of 1.0 and washed twice with RPMI 1640 × 10^6^ neutrophils were incubated with the bacteria at a ratio of 1:20 in HBSS with or without anti-PA14 serum (1:10 dilution) for 10 min. The extracellular bacteria were removed by centrifugation at 300*g* for 5 min. The supernatant was discarded and the cells were resuspended in 1 mL HBSS, followed by cytospin. The cells were imaged by confocal microscopy (Olympus FV1200).

#### Mouse acute pneumonia model

6-8 weeks female BALB/c mice were used in the infection experiments (8 mice per group). Before the infection, mice were anesthetized by intraperitoneal injection with 80 μL 7.5% chloral hydrate. Each mouse was inoculated intranasally with 20 μL PBS containing 4 × 10^6^ CFU of PA14. After 2 h, 200 μL neutrophil with or without azithromycin and CST-liposome were injected intravenously into the tail vein of each mouse. At 4 hpi, the mice were euthanized by inhalation of CO_2_, the lungs and blood were isolated and the drug concentration was measured by ELISA. Ten hours after the infection, the mice were euthanized by inhalation of CO_2_. The organs (lungs, hearts, livers and spleens) were homogenized and the bacterial counts were determined by plating. Alternatively, the survival of the mice was monitored at least twice every day for 5 days.

#### *In vivo* images

Neutrophils were incubated with DiR-labeled liposomes for 2 h, followed by washing with PBS to remove the free liposomes. Mice were infected intranasally with 4 × 10^6^ CFU of PA14 as aforementioned. 2 h post infection, the neutrophils were injected intravenously into the tail vein. 2 h after injection of the cells, the lungs were isolated and fixed by formaldehyde. The cryosections of the lungs were stained with a Ly6G antibody (Servicebio, Wuhan, China) and DAPI (Servicebio, Wuhan, China) and observed by microscopy.

#### Biological safety assay

Ten 6-8 weeks female BALB/c mice were divided into two groups, one group was injected with drug loaded neutrophils in the tail vein and the other group was injected with PBS as the control. The body weight of each mouse was measured every day. 14 days later, the mice were sacrificed by CO_2_. The heart, liver, spleen, lung and kidney were isolated, followed by H&E staining and observation by microscopy.

#### RNA extraction and quantitative real-time PCR

Neutrophils with or without azithromycin and colistin were incubated with PA14 (neutrophils:bacteria = 10:1). After 2 h, the cells were collected by centrifugation and resuspended in TRIzol (Thermo Fisher Scientific - USA). The total RNA was extracted by a Direct-zol RNA Miniprep kit (Zymo, USA). In the acute pneumonia model (5 mice per group), the organs (lungs, hearts, livers, kidneys and spleens) were isolated 10 h post infection, followed by homogenization in TRIzol. The total RNA was also extracted by a Direct-zol RNA Miniprep kit. Sequencing and analysis services were performed by the Suzhou Genewiz as previously described (Li et al., 2016).

cDNA was synthesized by a 5×mix PrimeScript Reverse Transcriptase (Vazyme, Nanjing, China). Quantitative real-time PCR was conducted using a CFX Connect Real-Time system (Bio-Rad, USA). The β-actin gene was used as an internal control.

#### Determination of cytokine levels by ELISA

Neutrophils with or without azithromycin and colistin were incubated with PA14 (neutrophils:bacteria = 10:1) in RPMI for 2 h. The supernatant was collected by centrifugation. In the acute pneumonia model (4 mice per group), the lungs were isolated 10 h post infection, followed by homogenization in PBS. The supernatant was collected by centrifugation. The IL-1β, IL-6 and TNF-α levels in the supernatants were determined by ELISA kits (Jiangsu Wise Science and Technology Development, China) according to the manufacturer’s instructions.

### Quantification and statistical analysis

All experiments were conducted at least 3 times. Statistical significance is reported in Figure Legends, and data are presented as mean ± SD as indicated. Bacterial killing was analyzed by Student’s *t* test. The quantitative real-time PCR and ELISA results were analyzed by two-way ANOVA. The mice survival results were analyzed with a log rank test with Prism software (Graphpad Software). The schematic illustrations in this report were created with the online BioRender software (https://biorender.com).

## Data Availability

Microscopy data reported in this paper will be shared by the lead contact upon request. The data generated from this study is deposited at the NCBI SRA database with the accession number SRA: PRJNA869576. Any additional information required to reanalyze the data reported in this paper is available from the lead contact upon request

## Supplemental information

Document S1. Figures S1–S5

Table S1. Antibiotic minimum inhibitory concentrations for the bacterial strains, related to Figure 4

Table S2. RNA-seq results of differently expressed genes, related to Figure 5d
