# Supplementary material for: Neutrophil-mediated delivery of the combination of colistin and azithromycin for the treatment of bacterial infection
Source: iScience. 2022 Aug 30;25(9):105035. doi: 10.1016/j.isci.2022.105035 (PMC9474925; doi:10.1016/j.isci.2022.105035)
Supplement: Document S1. Figures S1–S5 [file mmc1.pdf]

iScience, Volume *25*

Supplemental information

Neutrophil-mediated delivery of the combination of colistin and azithromycin for the treatment of bacterial infection

Jiacong Gao, Xueyan Hu, Congjuan Xu, Mingming Guo, Shouyi Li, Fan Yang, Xiaolei Pan, Fangyu Zhou, Yongxin Jin, Fang Bai, Zhihui Cheng, Zhenzhou Wu, Shuiping Chen, Xinglu Huang, and Weihui Wu

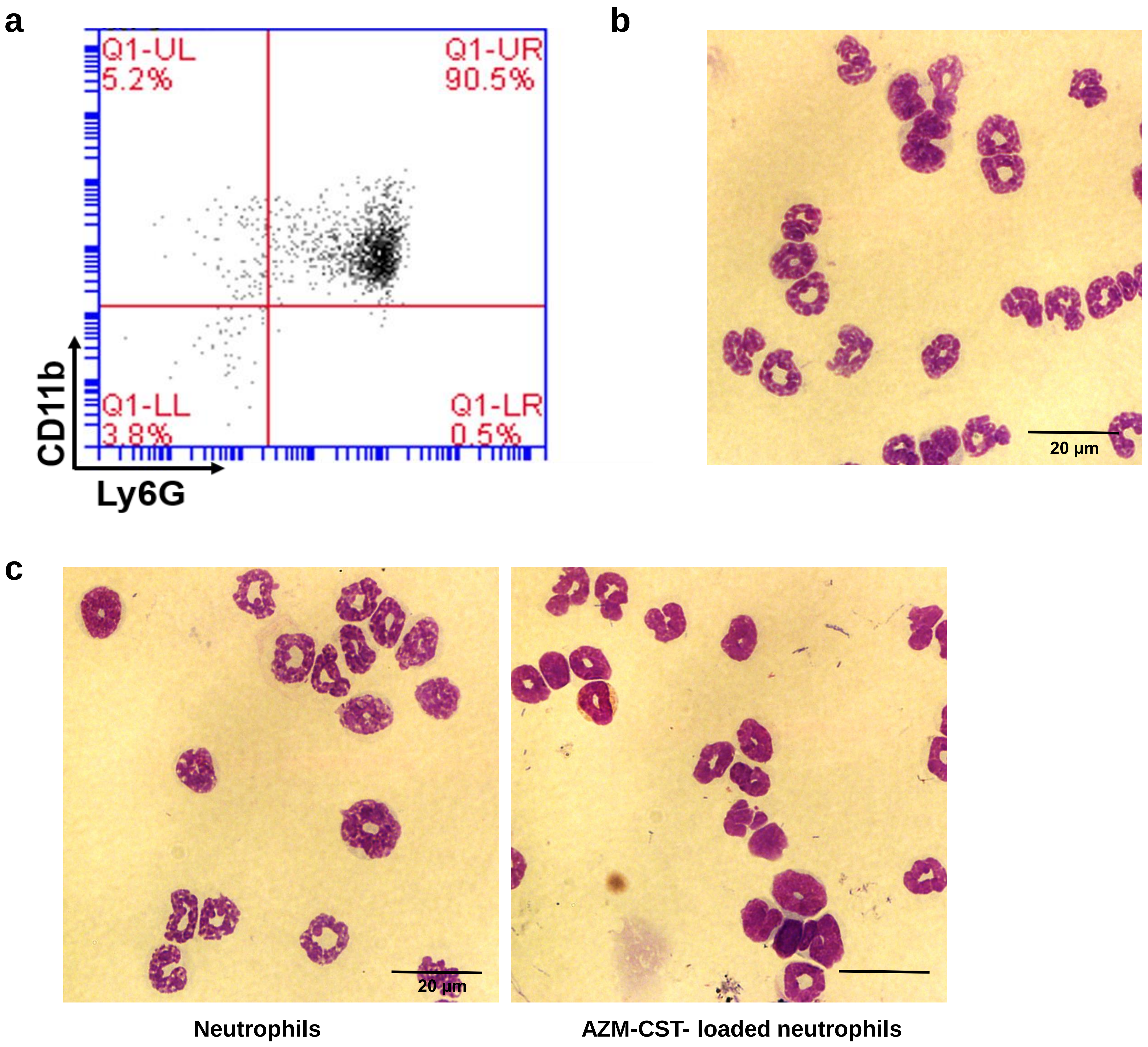

Figure S1. Isolation and characterization of neutrophils, Related to Figure 2 (a) Flow cytometry analysis of the purity of the isolated neutrophils. (b-c) Morphological images of isolated neutrophils (b) and drug loaded neutrophils (c) stained with Diff-Quik stain. Scale bar: 20 μm.

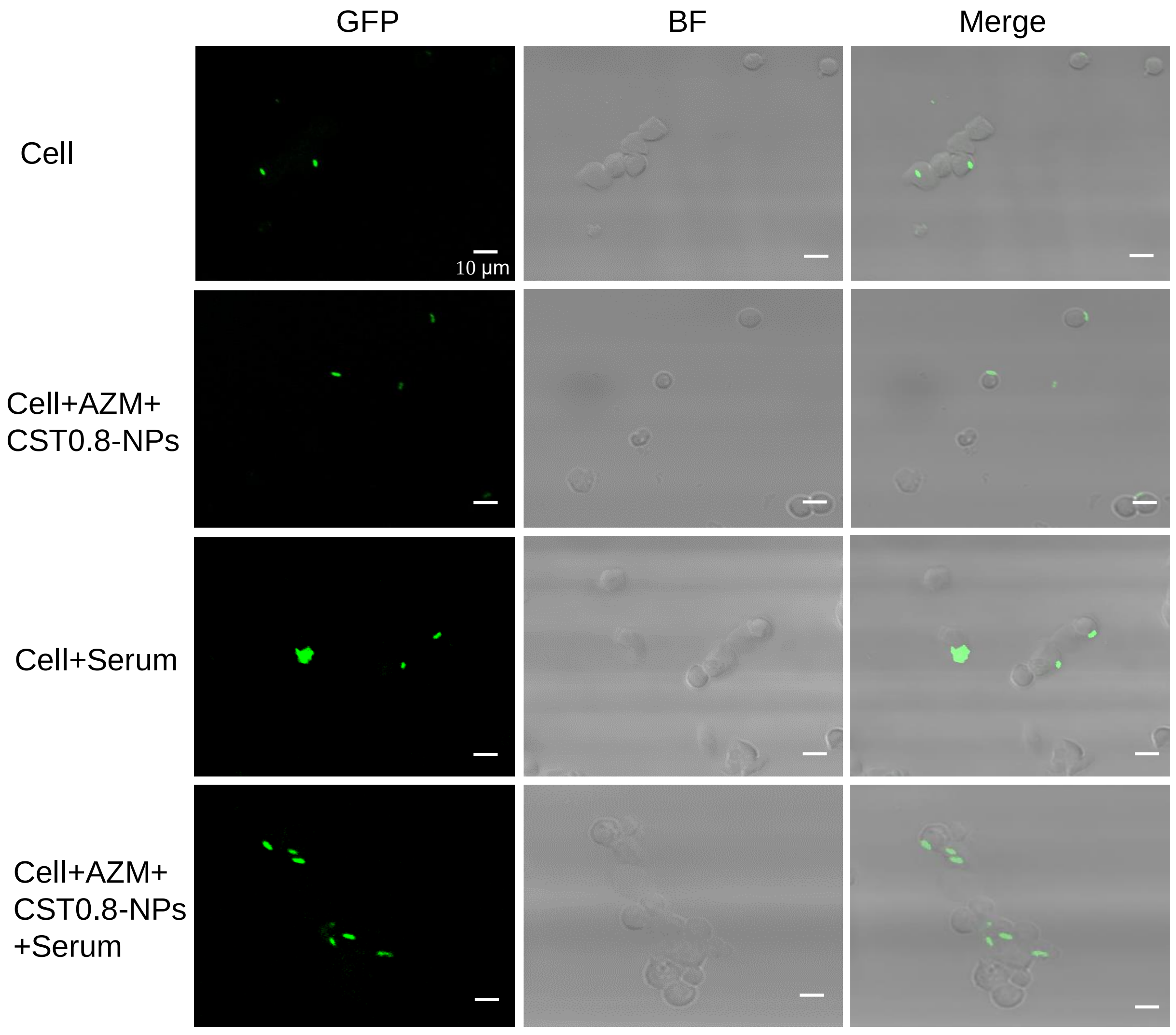

Figure S2. Phagocytosis ability of the drug loaded neutrophils, Related to Figure 5c. Fluorescent bacteria (PA14/pUCP20-gfp) were incubated with drug loaded and drug free neutrophils with or without the anti-PA14 serum (1:10 dilution) for 10 minutes. The uptaken bacteria were observed by confocal microscopy. Scale bar: 10 μm.

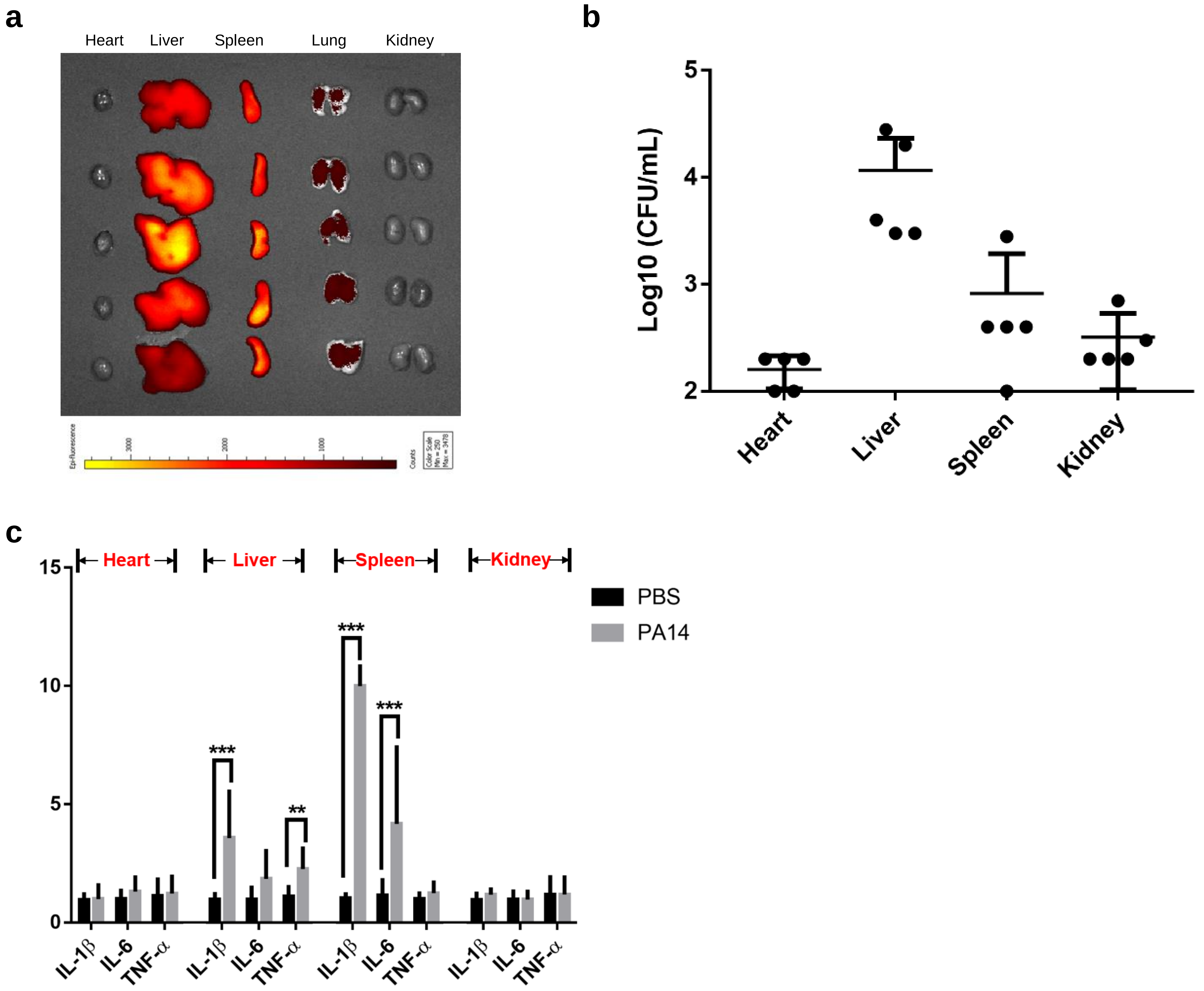

Figure S3. Biodistribution of administered drug-loaded neutrophils to infected mice, Related to Figure 6. (a) Fluorescence imaging of mice organs after intravenous injection of drug loaded neutrophils. (b) The bacterial loads in the organs (hearts, livers, spleens and kidneys) were determined at 10 hpi. (c) At 10 hpi, the organs (hearts, livers, spleens and kidneys) were isolated. The relative mRNA levels of genes encoding IL-1β, IL-6 and TNF-α were determined by real time PCR. **, $P<0.01$, ***, $P<0.001$ by Two-way ANOVA.

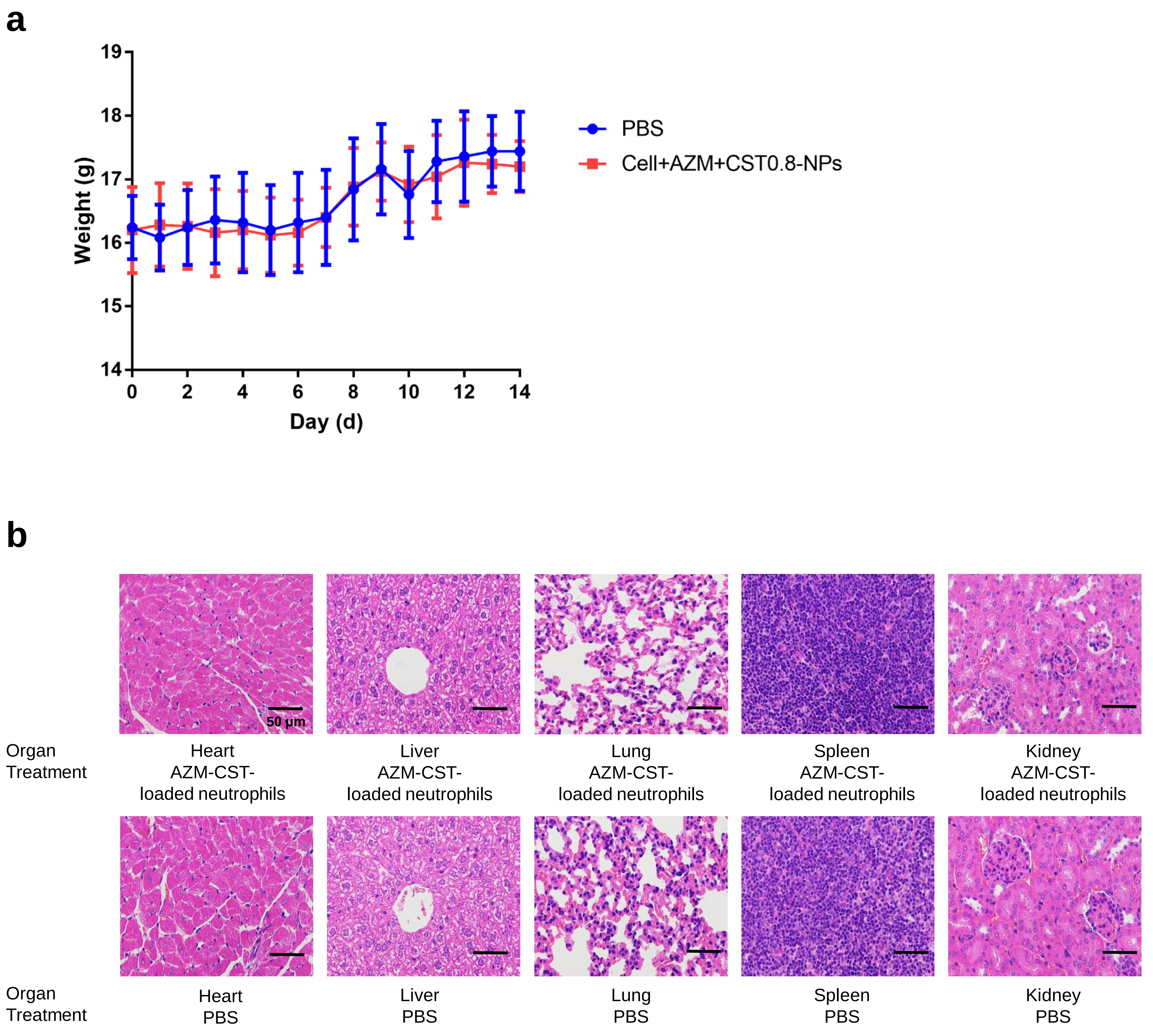

Figure S4. Effects of injection drug loaded neutrophils on the mice, Related to Figure 6. (a) Weight gain curves of mice following injection of PBS or drug loaded neutrophils. (b) Hematoxylin and eosin (H&E) staining of the mouse heart, livers, lung, spleen and kidney. Scale bar: 50 μm. PBS, phosphate buffered solution; AZM, azithromycin; CST, colistin.

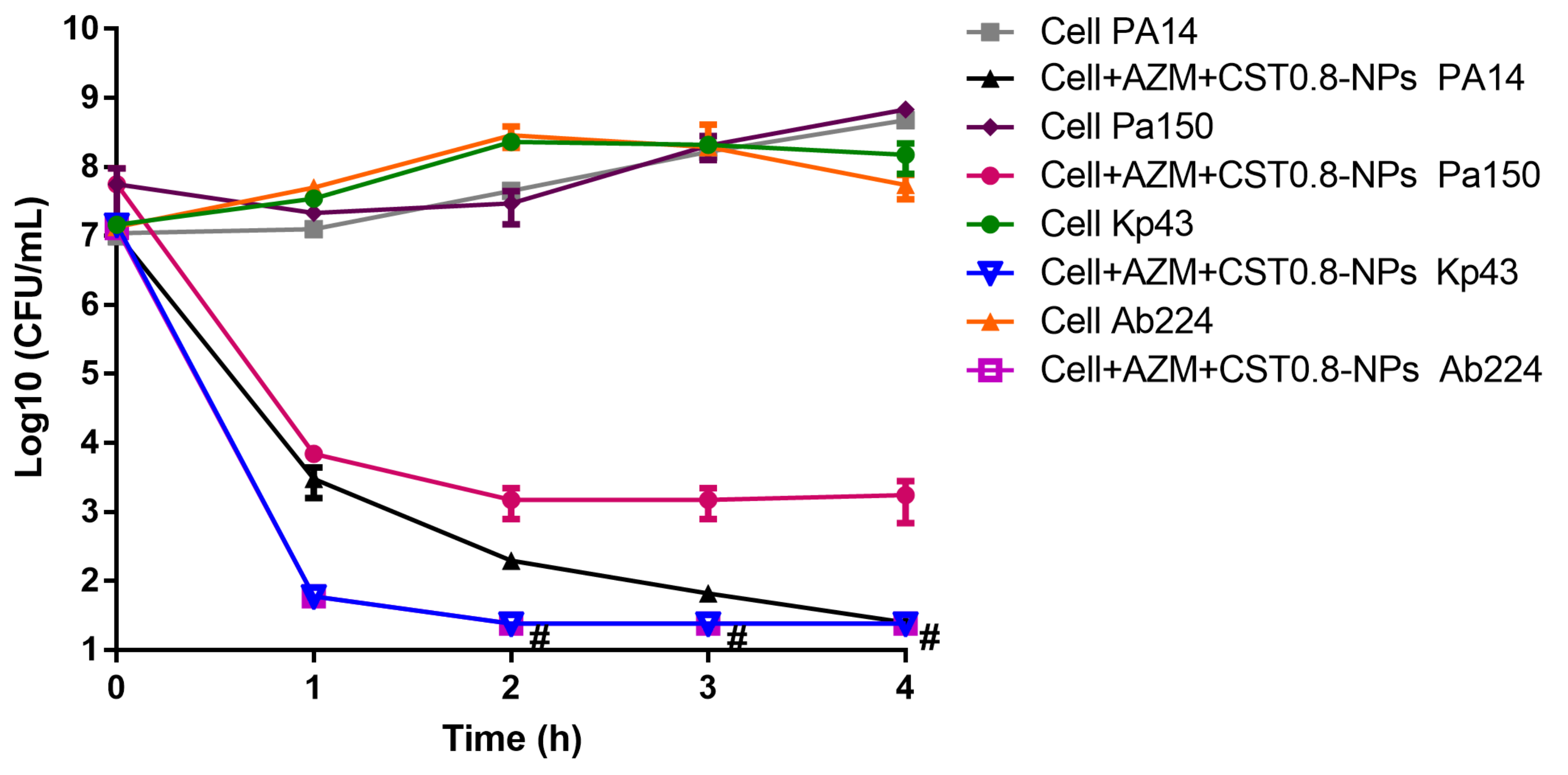

Figure S5. Bacteria killing efficacies of human peripheral blood neutrophils, Related to Figure 4. Time-kill curves of human peripheral blood neutrophils loaded with azithromycin and colistin against PA14, the MDR clinical isolates of *P*. *aeruginosa* (Pa150), *K*. *pneumoniae* (Kp43) and *A. baumannii* clinical isolates (Ab224). #, < detection limit (25 CFU/mL).
